# A Case of Papillary Thyroid Carcinoma in Struma Ovarii and Review of the Literature

**DOI:** 10.4061/2010/352476

**Published:** 2010-08-02

**Authors:** W. D. Salman, Mayuri Singh, Z. Twaij

**Affiliations:** Department of Histopathology, East Lancashire Hospitals NHS Trust, Burnley BB10 2PQ, UK

## Abstract

Malignancy in struma ovarii is a rare form of ovarian germ cell tumour. Because of its rarity, the diagnosis and management of the tumour have not been clearly defined. We present a case of 67- year-old female with papillary carcinoma arising in struma ovarii and review the literature on malignancy in struma ovarii cases, focusing on management of these cases.

## 1. Introduction

15%–20% of ovarian tumours are teratoma. Struma ovarii is diagnosed when thyroid tissue is the predominant element (>50%) [1]. 5%–10% of these tumours are malignant, with papillary carcinoma and follicular carcinoma being the most common [1–3]. The percentage of papillary thyroid carcinoma within malignant struma ovarii is 70%, 44% of the tumours being classical type and 26% follicular variant of papillary thyroid carcinoma [4]. Recently, a new entity of follicular carcinoma, highly differentiated follicular carcinoma of ovarian origin (HDFCO), characterized by extraovarian dissemination of thyroid elements and histological resemblance to nonneoplastic thyroid tissue has been described [5]. Due to the rarity of the disease, its treatment is not uniform. Here we present a rare case of struma ovarii with papillary thyroid carcinoma, and we review the management and treatment option of this rare tumour.

## 2. Case Report

A 67-year-old female was admitted with abdominal distension and rapidly developing ascites. Ultrasound examination and CT scan of the abdomen and pelvis showed extensive abdominopelvic ascites of unknown cause. No intra-abdominal mass or pelvic abnormality was detected. Tumour marker CA125 was raised, 2000 KU/l (normal—less than 35 KU/l) but serum CEA levels were within normal limits. Clinically, ovarian cancer was suspected, however paracentesis demonstrated benign peritoneal effusion. A transvaginal scan showed solid/cystic mass in the pouch of douglas 8 × 6 × 4 cm. She underwent laparotomy which showed copious amount of benign ascitic fluid and a left ovarian mass. The possibility of a dermoid cyst was considered. A total abdominal hysterectomy with bilateral salpingo-oopherectomy was performed along with omental biopsy and peritoneal washing.

**Figure 1:**
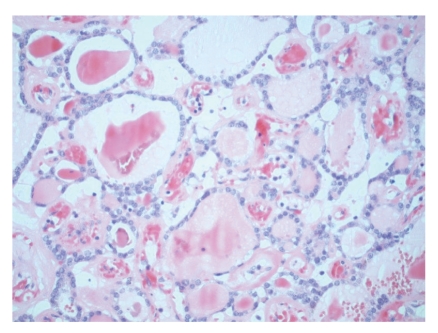
Low-magnification view of benign thyroid tissue in the ovary.

**Figure 2:**
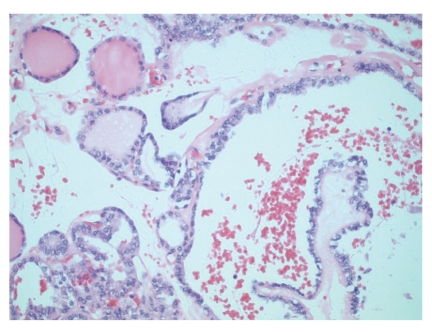
Areas with benign follicles as well as malignant papillary architecture.

**Figure 3:**
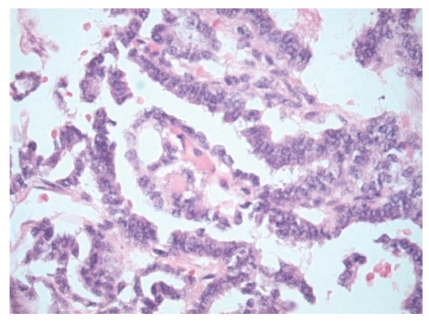
Papillary architecture with fibrovascular core.

**Figure 4:**
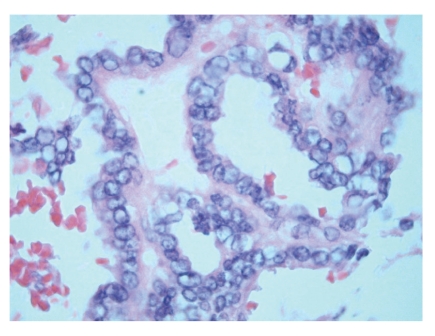
Areas with optically clear nuclei, thickened nuclear membrane, and overlapping nuclei representing papillary carcinoma.

**Figure 5:**
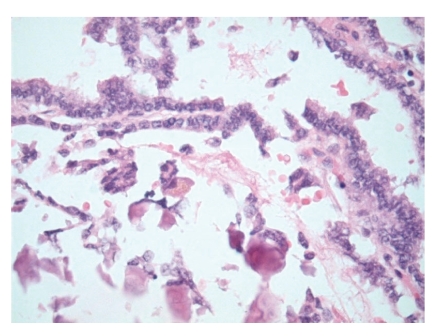
Areas with papillary structures and psamomma bodies.

On gross pathological examination, there was a left ovarian mass measuring 10 × 7 × 3.5 cm. The external surface of the cyst was mainly smooth with a small area of yellow/green discolouration. Cut section of the cyst showed haemorrhagic solid mass. Histology of the ovarian tumour showed thyroid tissue characteristic of struma ovarii (Figure 1). However, the thyroid tissue showed focal worrying features in the form of small and large papillae (Figures 2 and 3) lined by cells showing optically clear nuclei with thickened nuclear membrane and overlapping nuclei (Figure 4). Scattered psammoma bodies were also seen (Figure 5). The case was sent for second opinion. The final report confirmed it to be a struma ovarii with thyroid tissue showing neoplastic transformation into classical papillary thyroid carcinoma. The exact proportion and size of the carcinoma was difficult to estimate due to the smooth blending of the benign and malignant components. However, the overall malignant component was small measuring approximately 5 mm. Immunohistochemistry and molecular studies were not performed at our centre or by the histopathologist providing second opinion as the features were unequivocal of classical papillary thyroid carcinoma. The uterus, right ovary and the Fallopian tubes were unremarkable. The peritoneal washing and omental biopsy were negative for malignancy. Postoperative thyroid function test was within normal limits. Clinically there was no evidence of metastasis. The patient was staged as FIGO stage Ia malignant struma ovarii and no other adjuvant treatment was given. It was decided to keep her on follow-up for the next five years. Presently, after a two year follow-up, she is well with no evidence of recurrence.

## 3. Discussion

Struma ovarii is a rare and highly specialized form of mature teratoma constituting 5% of all teratomas. The age of presentation of malignancy in struma ovarii is usually in the 5th decade of life [1, 4]. CA 125 may be raised as seen in germ cell tumours. Ascites has been reported in 17% cases, but the fluid rarely contains tumour cells [4, 6]. 

A preoperative diagnosis of struma ovarii can be suspected in cases with hyperthyroidism, but this is seen only in 5%–8% cases [4]. On radiology, the possibility can be raised when a solid and cystic teratoma-like ovarian tumour shows a well-vascularized solid component on colour Doppler ultrasound, especially when a strongly enhancing solid component is found in a multilocular tumour of the ovary on computed tomography or MRI [7]. 

Macroscopically, the tumor is typically brown or green-brown, predominantly solid and gelatinous. The thyroid nature of this lesion has been confirmed with biologic and immunohistochemical studies [8]. Struma ovarii may demonstrate all the pathologic patterns that are seen in the thyroid gland including malignancy. The diagnosis of thyroid-type carcinoma arising in struma ovarii largely depends on the recognition of its characteristic microscopic features with hematoxylin eosin-stained sections. The criteria for malignancy was reviewed by Devaney et al. and features is similar to that seen in thyroid, such as ground-glass overlapping nuclei, nuclear grooves, and papillary architecture for papillary thyroid carcinoma in struma ovarii. Lesions showing hyperplastic type papillary formations but lacking nuclear features have been diagnosed as proliferative struma [1]. Lesions with nuclear features of papillary thyroid carcinoma but lacking papillary architecture represent follicular variant of papillary thyroid carcinoma (FVPTC). The diagnosis of well-differentiated thyroid type follicular carcinoma is more difficult. Capsular invasion is an important criterion of malignancy in follicular carcinoma, but there is usually no capsule in the corresponding ovarian lesion. Therefore, the identification of invasion into the surrounding ovarian tissue, vascular invasion, or metastasis is employed as evidence of malignancy [3]. The less differentiated forms show significant architectural abnormalities, nuclear atypia, and mitotic activity. Immunohistochemical markers have been suggested to help in the distinction between benign thyroid tissue and papillary carcinoma of the thyroid, Cytokeratin 19, HBME-1, and galectin-3 have been reported to be valuable for this purpose [9, 10]. Roth and Karseladze have recently suggested the term highly differentiated follicular carcinoma of ovarian origin (HDFCO) for tumours resembling nonneoplastic thyroid tissue in both the ovary and sites of dissemination and proposed this term for peritoneal strumosis cases. Because of the benign histologic appearance of HDFCO, this form of follicular carcinoma cannot be diagnosed until the neoplasm spreads beyond the ovary [5]. 

Thyroid type carcinoma can also be seen in a mature cystic teratoma or can be a component of strumal carcinoid. Strumal carcinoid is a form of ovarian teratoma characterized by a mixture of thyroid tissue and carcinoid. Immunohistochemistry using TTF-1 (thyroid transcription factor 1), thyroglobulin, and neuroendocrine markers, such as chromogranin or synaptophysin may assist in the diagnosis [3]. The cases of strumal carcinoid with a component of thyroid-type carcinoma should be diagnosed as thyroid-type carcinoma to ensure patients receive optimal follow-up and therapy [11].

Malignancy arising in struma ovarii may mimic other primary ovarian tumors, such as granulosa cell tumor, Brenner tumor, papillary serous cystadenoma or cystadenocarcinoma. Granulosa tumor or Brenner tumor can be a component of mature cystic teratoma and may have a microfollicular or pseudotubular appearance with grooved nuclei, which may simulate follicular carcinoma or follicular-variant papillary thyroid carcinoma. The papillary appearance and the presence of psammoma bodies in ovarian papillary serous cystadenoma or cystadenocarcinoma may mimic thyroid-type papillary carcinoma. In such cases diagnosis can be made by the cytologic features of the neoplastic cells, the presence of typical thyroid follicles and immunohistochemistry such as thyroglobulin, TTF-1, inhibin, WT1 (Wilms tumor 1), and CA 125 will help differentiate these ovarian primary tumors from thyroid-type carcinoma [3, 11, 12].

 Molecular analysis has revealed that approximately 70% of all follicular cell–derived thyroid carcinomas present with activating mutations of BRAF (v-raf murine sarcoma viral oncogene homolog B1), RAS, RET (rearranged during transfection) and NTRK1 (neurotrophic tyrosine kinase receptor 1) [13–15].* BRAF* mutations are common in papillary thyroid carcinoma and are seen in two-thirds of malignant struma ovarii with papillary features as described by Schmidt et al. BRAF mutations included V600E, K601E, and TV599-600M [16]. Flavin et al. described a case of classical papillary thyroid carcinoma arising in a struma ovarii with heterozygous for BRAF T1799A mutation and no ret/ PTC-1 or ret/PTC-3 rearrangements [17]. Kondo et al. reviewed the pathogenetic mechanisms of thyroid follicular cell neoplasia and found mutations of BRAF (29%–69%), RET (13%–43%), and RAS (0%–21%) are most commonly seen in adult papillary thyroid carcinoma; RET rearrangements are more prevalent in adult tumors associated with previous radiation exposure [14]. Celestino et al. reported a case of follicular variant of papillary thyroid carcinoma in a struma ovarii with NRAS mutation (Q61R) and a PAX8-PPARc rearrangement which fitted well with the similar results seen in cervical counterpart [18] and Coyne and Nikiforov reported HRAS codon 61 mutation in a case of follicular variant of papillary thyroid carcinoma in a struma ovarii [19]. Papillary carcinomas harboring RAS mutation almost always have the follicular variant histology. Boutross-Tadross et al. examined 10 cases of follicular variant papillary thyroid carcinoma in struma ovarii and 3 cases of benign struma ovarii and found all of the carcinomas were diffusely positive for CK19 (cytokeratin 19), 8 were positive for HMBE-1 (hector battifora mesothelial cell 1), and 7 exhibited RET/PTC rearrangement (ret/PTC-1 and ret/PTC-3 rearrangements). Mutational analysis for BRAF identified no V600E mutations. All 3 benign struma ovarii were negative for CK19, HBME-1, and RET/PTC rearrangement [20]. These molecular findings suggest that thyroid-type carcinoma in struma ovarii are similar histologically and genetically to cervical thyroid carcinoma.

Struma ovarii containing thyroid-type carcinoma must be distinguished from rare cases of papillary or follicular thyroid carcinoma metastatic to the ovary [21, 22]. Metastasis to the ovary from primary thyroid carcinoma can be ruled out by clinical thyroid examination and ultrasonography. In these cases, the ovarian masses are bilateral and have no teratomatous features [23]. 

Metastasis is uncommon in patients with malignant struma ovarii, seen in 5% to 23% cases [4]. The potential of recurrence and metastasis was considered low in the previous literatures [1]. However, the recent literatures suggest a higher rate of recurrence [4, 6, 24]. Roth et al. [25] reviewed their own cases as well as literature cases and described that a typical follicular carcinoma is more likely to metastasize to the lung, liver, and central nervous system; whereas papillary carcinoma involve the abdominal cavity and lymph nodes and occasionally the liver [25]. 

The management of cases of struma ovarii with thyroid type malignancy is based on case reports and small cases series review. Devaney et al. studied 54 cases of struma which were subdivided into “proliferative” struma (41 cases) and “malignant” struma (13 cases). 11 of the 13 were papillary carcinomas of thyroid type, whereas 2 were follicular carcinoma. None of the patients received adjuvant therapy. On follow-up examination (mean follow-up interval 7.3 years), none of the patients had clinical evidence of recurrent disease [1]. 

DeSimone et al. reviewed the literature on malignancy in struma ovarii in a series of 24 patients. 16 patients were followed conservatively, while 8 received varied additional therapy (4 cases received I^131^). There were 8 cases of recurrences which occurred in the conservatively managed patients. I^131^ for recurrent disease provided an initial complete response in 7 women. Therefore, they suggest treatment with thyroidectomy and I^131^ as the first line of management for malignant struma ovarii [6].

Surgical removal of the ovarian mass remains the main treatment; however the management after initial surgery is still controversial. Mattucci et al. suggest the management of malignancy in struma ovarii should be the same as for carcinoma of the thyroid, so after surgical removing of ovarian neoplasm, they recommend thyroidectomy, radiotherapy with ^131^I, and levothyroxine suppressive therapy [26].

Makani et al. reviewed all reported cases till 2004, a total of 39 cases. They found metastasis in nine cases (23%) and recurrence in six cases (15%). The average time to detection of recurrence was four years [4]. They recommend follow-up with surveillance of thyroglobulin levels for at least 10 years. Thyroglobulin is a sensitive marker for monitoring cases of struma ovarii, both benign and malignant, during treatment and follow-up [2, 27, 28]. 

Ozata et al. described that 98% of thyroid cancer patients with a serum thyroglobulin less than10 ng/ml were clinically free of disease [29]. Therefore, some authors suggest initiating ^131^I therapy in patients with serum thyroglobulin of >10 ng/mL. For detecting recurrence, serum thyroglobulin and serial ^131^I diagnostic whole body scanning is suggested. In patients with elevated thyroglobulin who do not respond to radioactive iodine, PET/CT is considered most useful in the detection and management of recurrent papillary thyroid cancer [30].

Some authors have advocated the management of malignancy in struma as other germ cell tumours [31] while others have proposed that it should be treated like its thyroid counterpart. The latter is the favoured approach in the recent literatures [32–34].

The standard treatment of a patient with thyroid malignancy in struma ovarii is total abdominal hysterectomy, bilateral salpingo-oophorectomy, and complete surgical staging, including peritoneal washings for cytology, pelvic and para-aortic lymph node sampling, and omentectomy [4, 28]. In cases with residual malignant disease after surgery, a total thyroidectomy and radioablation with ^131^I is recommended [6, 35]. Chemotherapy and external beam radiotherapy and thyroid suppression have been used for the treatment of recurrent or metastatic disease [26]. 

Yassa et al. suggest a risk stratification of malignancy in struma ovarii patients; small focus of thyroid carcinoma confined to the struma ovarii measuring less than 2 cm, with no worrisome histologic features to be considered as low risk. Patients with larger carcinomas, disease outside the struma ovarii, or more aggressive histologic features are considered as high risk. For younger patients with malignant struma ovarii who wish to preserve fertility, oophorectomy is appropriate surgery if there is no extra-ovarian disease. For patients with low risk of persistent or recurrent thyroid carcinoma, thyroxine therapy, pelvic imaging, and periodic measurements of serum thyroglobulin are recommended and in patients with a higher risk of recurrence based on the pathology of the carcinoma, near-total thyroidectomy with radioactive iodine ablation is indicated [24].

Janszen et al. recommend that the best option for patients with malignant struma ovarii larger than one cm is total thyroidectomy followed by ^131^I ablation therapy. After ^131^I ablation any detectable serum thyroglobulin points to persistent or recurrent disease [32].

The prognosis of thyroid-type carcinoma arising in struma ovarii is difficult to estimate due to its rarity and the absence of consensus in treatment. Roth et al. reviewed the literature and revealed 14% with typical follicular carcinoma, 7% with papillary carcinoma, 100% with undifferentiated (anaplastic) carcinoma, and 0% with HDFCO died of neoplasm [25]. Robboy et al. reviewed 88 cases of malignant struma ovarii and they found that even when clinically malignant, the tumour is often associated with long survival, as evidenced by an 84% 25-year survival. They describe that unless obviously poorly differentiated, no single histologic or clinical feature reliably predicts which tumors will be biologically malignant, although dense fibrous adhesions and larger strumal size, especially over 12 cm, are suggestive of tumours that will have spread at the time of operation or are likely to recur [12]. 

In our case the focus of papillary thyroid carcinoma was small and the postoperative thyroid function test was normal. In the multidisciplinary meeting, it was decided that because the chance of recurrence was low, the patient shall be followed up.

In conclusion, the treatment modalities for malignancy in struma ovarii depend on the stage of the disease. The initial surgery options include unilateral oophorectomy; total hysterectomy and bilateral salpingo-oophorectomy or total hysterectomy, bilateral salpingooophorectomy with omentectomy and lymph node sampling. The adjuvant treatment options include thyroxine, near-total thyroidectomy with radioactive iodine ablation or no adjuvant treatment. Long-term follow-up is recommended in all cases.

## References

[1] Devaney K, Snyder R, Norris HJ, Tavassoli FA (1993). Proliferative and histologically malignant struma ovarii: a clinicopathologic study of 54 cases. *International Journal of Gynecological Pathology*.

[2] Kostoglou-Athanassiou I, Lekka-Katsouli I, Gogou L, Papagrigoriou L, Chatonides I, Kaldrymides P (2002). Malignant struma ovarii: report of a case and review of the literature. *Hormone Research*.

[3] Roth LM, Talerman A (2007). The enigma of struma ovarii. *Pathology*.

[4] Makani S, Kim W, Gaba AR (2004). Struma Ovarii with a focus of papillary thyroid cancer: a case report and review of the literature. *Gynecologic Oncology*.

[5] Roth LM, Karseladze AI (2008). Highly differentiated follicular carcinoma arising from struma ovarii: a report of 3 cases, a review of the literature, and a reassessment of so-called peritoneal strumosis. *International Journal of Gynecological Pathology*.

[6] DeSimone CP, Lele SM, Modesitt SC (2003). Malignant struma ovarii: a case report and analysis of cases reported in the literature with focus on survival and I131 therapy. *Gynecologic Oncology*.

[7] Van de Moortele K, Vanbeckevoort D, Hendrickx S (2003). Struma ovarii: US and CT findings. *Journal Belge de Radiologie*.

[8] Hasleton PS, Kelehan P, Whittaker JS, Burslem RW, Turner L (1978). Benign and malignant struma ovarii. *Archives of Pathology and Laboratory Medicine*.

[9] Cheung CC, Ezzat S, Freeman JL, Rosen IB, Asa SL (2001). Immunohistochemical diagnosis of papillary thyroid carcinoma. *Modern Pathology*.

[10] Weber KB, Shroyer KR, Heinz DE, Nawaz S, Said MS, Haugen BR (2004). The use of a combination of galectin-3 and thyroid peroxidase for the diagnosis and prognosis of thyroid cancer. *American Journal of Clinical Pathology*.

[11] Zhang X, Axiotis C (2010). Thyroid-type carcinoma of struma ovarii. *Archives of Pathology and Laboratory Medicine*.

[12] Robboy SJ, Shaco-Levy R, Peng RY (2009). Malignant struma ovarii: an analysis of 88 cases, including 27 with extraovarian spread. *International Journal of Gynecological Pathology*.

[13] Ciampi R, Nikiforov YE (2007). RET/PTC rearrangements and BRAF mutations in thyroid tumorigenesis. *Endocrinology*.

[14] Kondo T, Ezzat S, Asa SL (2006). Pathogenetic mechanisms in thyroid follicular-cell neoplasia. *Nature Reviews Cancer*.

[15] Xing M (2005). BRAF mutation in thyroid cancer. *Endocrine-Related Cancer*.

[16] Schmidt J, Derr V, Heinrich MC (2007). BRAF in papillary thyroid carcinoma of ovary (struma ovarii). *American Journal of Surgical Pathology*.

[17] Flavin R, Smyth P, Crotty P (2007). BRAF T1799A mutation occurring in a case of malignant struma ovarii. *International Journal of Surgical Pathology*.

[18] Celestino R, Magalhães J, Castro P (2009). A follicular variant of papillary thyroid carcinoma in struma ovarii. Case report with unique molecular alterations. *Histopathology*.

[19] Coyne C, Nikiforov YE (2010). RAS mutation-positive follicular variant of papillary thyroid carcinoma arising in a struma ovarii. *Endocrine Pathology*.

[20] Boutross-Tadross O, Saleh R, Asa SL (2007). Follicular variant papillary thyroid carcinoma arising in struma ovarii. *Endocrine Pathology*.

[21] Logani S, Baloch ZW, Snyder PJ, Weinstein R, LiVolsi VA (2001). Cystic ovarian metastasis from papillary thyroid carcinoma: a case report. *Thyroid*.

[22] Young RH, Jackson A, Wells M (1994). Ovarian metastasis from thyroid carcinoma 12 years after partial thyroidectomy mimicking struma ovarii: report of a case. *International Journal of Gynecological Pathology*.

[23] Brogioni S, Viacava P, Tomisti L, Martino E, Macchia E (2007). A special case of bilateral ovarian metastases in a woman with papillary carcinoma of the thyroid. *Experimental and Clinical Endocrinology and Diabetes*.

[24] Yassa L, Sadow P, Marqusee E (2008). Malignant struma ovarii. *Nature Clinical Practice Endocrinology and Metabolism*.

[25] Roth LM, Miller AW, Talerman A (2008). Typical thyroid-type carcinoma arising in struma ovarii: a report of 4 cases and review of the literature. *International Journal of Gynecological Pathology*.

[26] Mattuci ML, Dellera A, Guerriero A, Barbieri F, Minelli L, Furlani L (2007). Malignant struma ovarii: a case report and review of the literature. *Journal of Endocrinological Investigation*.

[27] Rose PG, Arafah B, Abdul-Karim FW (1998). Malignant struma ovarii: recurrence and response to treatment monitored by thyroglobulin levels. *Gynecologic Oncology*.

[28] Matsuda K, Maehama T, Kanazawa K (2001). Malignant struma ovarii with thyrotoxicosis. *Gynecologic Oncology*.

[29] Ozata M, Suzuki S, Miyamoto T, Liu RT, Fierro-Renoy F, DeGroot LJ (1994). Serum thyroglobulin in the follow-up of patients with treated differentiated thyroid cancer. *Journal of Clinical Endocrinology and Metabolism*.

[30] Hatami M, Breining D, Owers RL, Del Priore G, Goldberg GL (2008). Malignant struma ovarii—a case report and review of the literature. *Gynecologic and Obstetric Investigation*.

[31] Ayhan A, Yanik F, Tuncer R, Tuncer ZS, Ruacan S (1993). Struma ovarii. *International Journal of Gynecology and Obstetrics*.

[32] Janszen EWM, Van Doorn HC, Ewing PC (2008). Maligne struma ovarii. *Nederlands Tijdschrift voor Geneeskunde*.

[33] Dardik RB, Dardik M, Westra W, Montz FJ (1999). Malignant struma ovarii: two case reports and a review of the literature. *Gynecologic Oncology*.

[34] Vadmal MS, Smilari TF, Lovecchio JL, Klein IL, Hajdu SI (1997). Diagnosis and treatment of disseminated struma ovarii with malignant transformation. *Gynecologic Oncology*.

[35] Willemse PHB, Oosterhuis JW, Aalders JG (1987). Malignant struma ovarii treated by ovariectomy, thyroidectomy, and 131I administration. *Cancer*.

